# The complete mitochondrial genome of *Scutopus ventrolineatus* (Mollusca: Chaetodermomorpha) supports the Aculifera hypothesis

**DOI:** 10.1186/s12862-014-0197-9

**Published:** 2014-09-25

**Authors:** David Osca, Iker Irisarri, Christiane Todt, Cristina Grande, Rafael Zardoya

**Affiliations:** Department of Biodiversity and Evolutionary Biology, Museo Nacional de Ciencias Naturales, CSIC, José Gutiérrez Abascal 2, 28006 Madrid, Spain; Current address: Department of Biology, Laboratory for Zoology and Evolutionary Biology, University of Konstanz, Universitätsstr. 10, 78464 Konstanz, Germany; University Museum of Bergen, The Natural History Collections, University of Bergen, P.O. Box 7800, , NO-5020 Bergen, Norway; Departamento de Biología Molecular and Centro de Biología Molecular “Severo Ochoa” (CSIC - Universidad Autónoma de Madrid), Universidad Autónoma de Madrid, 28049 Madrid, Spain

**Keywords:** Mitogenomics, Nuclear ribosomal proteins, Caudofoveata, Aculifera, Aplacophora

## Abstract

**Background:**

With more than 100000 living species, mollusks are the second most diverse metazoan phylum. The current taxonomic classification of mollusks recognizes eight classes (Neomeniomorpha, Chaetodermomorpha, Polyplacophora, Monoplacophora, Cephalopoda, Gastropoda, Bivalvia, and Scaphopoda) that exhibit very distinct body plans. In the past, phylogenetic relationships among mollusk classes have been contentious due to the lack of indisputable morphological synapomorphies. Fortunately, recent phylogenetic analyses based on multi-gene data sets are rendering promising results. In this regard, mitochondrial genomes have been widely used to reconstruct deep phylogenies. For mollusks, complete mitochondrial genomes are mostly available for gastropods, bivalves, and cephalopods, whereas other less-diverse lineages have few or none reported.

**Results:**

The complete DNA sequence (14662 bp) of the mitochondrial genome of the chaetodermomorph *Scutopus ventrolineatus* Salvini-Plawen, 1968 was determined. Compared with other mollusks, the relative position of protein-coding genes in the mitochondrial genome of *S. ventrolineatus* is very similar to those reported for Polyplacophora, Cephalopoda and early-diverging lineages of Bivalvia and Gastropoda (Vetigastropoda and Neritimorpha; but not Patellogastropoda). The reconstructed phylogenetic tree based on combined mitochondrial and nuclear sequence data recovered monophyletic Aplacophora, Aculifera, and Conchifera. Within the latter, Cephalopoda was the sister group of Gastropoda and Bivalvia + Scaphopoda.

**Conclusions:**

Phylogenetic analyses of mitochondrial sequences showed strong among-lineage rate heterogeneity that produced long-branch attraction biases. Removal of long branches (namely those of bivalves and patellogastropods) ameliorated but not fully resolved the problem. Best results in terms of statistical support were achieved when mitochondrial and nuclear sequence data were concatenated.

**Electronic supplementary material:**

The online version of this article (doi:10.1186/s12862-014-0197-9) contains supplementary material, which is available to authorized users.

## Background

Mollusks are the second largest animal phylum with more than 100000 described extant species and are grouped into eight classes: Solenogastres or Neomeniomorpha, Caudofoveata or Chaetodermomorpha, Polyplacophora, Monoplacophora, Bivalvia, Gastropoda, Cephalopoda, and Scaphopoda [1–3]. Morphology-based classifications consider Neomeniomorpha and Chaetodermomorpha as the earliest branching lineages within mollusks because they lack many typical features of mollusks, among which the most conspicuous is the shell [4]. Whether they form a monophyletic group named Aplacophora [5,6] or a paraphyletic grade [7–10] is still a matter of debate [11]. Some authors [12–14] place Polyplacophora (chitons) as sister group of Conchifera (Monoplacophora, Bivalvia, Gastropoda, Cephalopoda, and Scaphopoda), forming the clade Testaria. Alternatively, the Aculifera hypothesis proposes a sister group relationship of Polyplacophora and Aplacophora, suggesting that the aplacophoran morphology was secondarily modified from a chiton-like ancestor [5,6,15,16]. Phylogenetic relationships within Conchifera are also far from settled due to the highly derived morphologies within each class-level grade, which hinder the discovery of morphological synapomorphies across lineages [17]. The traditional morphology-based hypothesis groups together Bivalvia + Scaphopoda (Diasoma or Loboconcha) and Cephalopoda + Gastropoda (Cyrtosoma or Visceroconcha) [3,9].

Earlier molecular studies based on partial sequences of one or few genes revealed important phylogenetic inference biases, and failed to recover the monophyly of mollusks and/or of several main lineages within the group [18]. The first studies analyzing the relative phylogenetic position of Monoplacophora [19,20] also rendered a surprising result in recovering the group as closely related to Polyplacophora, forming the taxon Serialia, which is in disagreement with most morphological evidence [21]. More recently, attempts to reconstruct the phylogeny of mollusks were based on concatenated matrices spanning many genes. A study [22] based on 79 ribosomal protein genes recovered the monophyly of the phylum and of all five mollusk classes included in the analyses (Neomeniomorpha, Monoplacophora, and Scaphopoda were missing). However, recovered interclass relationships, although highly supported, were rather unconventional, with Bivalvia and Gastropoda being sister group to Polyplacophora, and this clade being sister group to Cephalopoda and Chaetodermomorpha [22]. The latest phylogenetic studies based on seven housekeeping genes [23], and on genomic-scale data sets spanning 308 [24] and 1185 [25] genes, respectively, recovered monophyletic Mollusca, Aplacophora, and Aculifera, thus rejecting the Testaria hypothesis. One of these studies included Monoplacophora, which was placed within Conchifera, thus providing no support for the Serialia hypothesis [25]. Interestingly, phylogenomic studies [24,25] arrived at highly supported but contrasting conchiferan interclass relationships. While both studies support a basal position of Cephalopoda (+Monoplacophora in [25]), one favors a clade composed of Gastropoda and Bivalvia (the so-called Pleistomollusca [24]) whereas the other groups together Gastropoda and Scaphopoda [25].

During the last decade, complete mitochondrial (mt) genomes have become a standard for phylogenetic reconstruction of animal relationships [26]. Although the number of completely sequenced mollusk mt genomes has increased considerably in the last few years, the majority belong to the most common and economically important mollusk classes i.e., Cephalopoda [27], Bivalvia [28], and Gastropoda [29,30]. In addition, there are reported three Polyplacophora, *Katharina tunicata* [31] and two *Sypharochiton* species [32], and two Scaphopoda, *Siphonodentalium lobatum* [33] and *Graptacme eborea* [34] mitogenomes (but note that the mt genome of a Chaetodermomorpha, *Chaetoderma nitidulum* is available at NCBI, although unpublished). The only published study [35] that has applied thus far whole mt genome data for reconstructing phylogenetic relationships of mollusks failed to recover the monophyly of Mollusca and of many mollusk classes due to long branch attraction (LBA) artifacts. The authors concluded that representatives from all mollusks classes and a denser taxon sampling of most diverse lineages could render a more resolved mollusk interclass phylogeny, and that mt gene order data could become a promising source of phylogenetic information [35].

In this paper, we present the complete mitochondrial genome of *Scutopus ventrolineatus*, a representative of the supposedly early-branching Limifossoridae within Chaetodermomorpha. We performed comparative genomic analyses with other available mollusk mt genomes with the specific aim of addressing the evolution of gene order arrangements among the main lineages within the phylum. In addition, we used generated mt sequence data to infer phylogenetic relationships of mollusks, and in particular to test the validity of traditional morphology-based hypotheses that place Chaetodermomorpha at the base of the mollusk tree. Finally, we concatenated mt genomes with publicly available nuclear sequence data in trying to maximize statistical support of the reconstructed mollusk phylogeny.

## Results and discussion

### Mitochondrial genome organization and structural features

**Figure 1:**
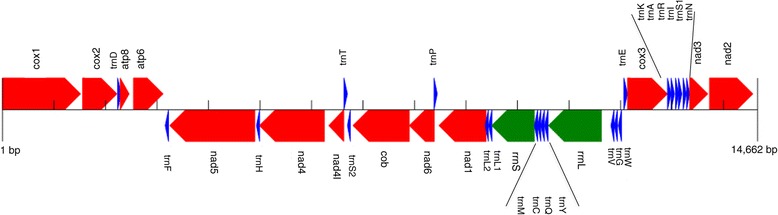
**Mitochondrial gene order of** ***Scutopus ventrolineatus.*** Protein-coding (red), rRNA (green) and tRNA (blue) genes are shown. Arrows indicate sense of transcription. Genes have standard abbreviations and are not scaled to real length. S1, S2, L1, and L2 designate genes for those tRNAs recognizing the codons AGN, UCN, CUN, and UUR, respectively. Figure modified from the output of the MITOS pipeline [44].

The complete mt genome of *Scutopus ventrolineatus* was assembled as a 14662 bp circular molecule. Like most metazoan mt genomes, it encodes for 13 protein-coding, 22 tRNA and 2 rRNA genes (Figure 1). The major strand encodes 20 out of the 37 genes (*trnF, nad5, trnH, nad4, nad4L, trnS(UCN), cob, nad6, nad1, trnL(UUR), trnL(CUN), rrnS, trnM, trnC, trnQ, trnY, rrnL, trnV, trnG, trnW*). Most protein-coding genes start with the codon ATG with the exception of *atp8*, which begins with GTG. Several genes show complete stop codons, either TAA (as in *cox1*, *atp8*, *atp6*, *nad4l*, *cob*, and *nad6*) or TAG (as in *cox2* and *nad1*). The remaining genes finish with either TA (*nad5*) or a single T (*cox3*, *nad2*, *nad4*), which presumably become functional stop codons by subsequent polyadenylation of the transcribed messenger RNAs [36]. Four genes overlap with contiguous genes: *cox2* with *trnD*; *atp8* with *atp6*; *nad4* with *trnH*; and *nad4L* with *nad4*. The largest noncoding region has 47 bp, and it is located between *trnW* and *trnE* genes.

The gene order arrangement of the mt genome of *S. ventrolineatus* was compared with the mt genome organization in other mollusks. The unpublished mt genome of the chaetodermomorph *C. nitidulum* is the closest to compare, but it is 40% longer (21008 bp), and shows a duplication of the *cox2* gene, as well as large non-coding regions (see Additional file 1: Figure S1). In addition, several tRNA genes (*trnV*, *trnF*, *trnG*, *trnW*, *trnA*, *trnR*, *trnI*, *trnS (UCN)*) are reordered and two (*trnV* and *trnS (UCN)*) are encoded on opposite strands when compared to the mt genome of *S. ventrolineatus* (see Additional file 1: Figure S1). Since the mt genome organization of *S. ventrolineatus* conforms to the mollusk consensus gene order (see below), it would be important to determine the origin of the highly divergent mt genome of *C. nitidulum*, and discard putative sequencing or assembly errors.

**Figure 2:**
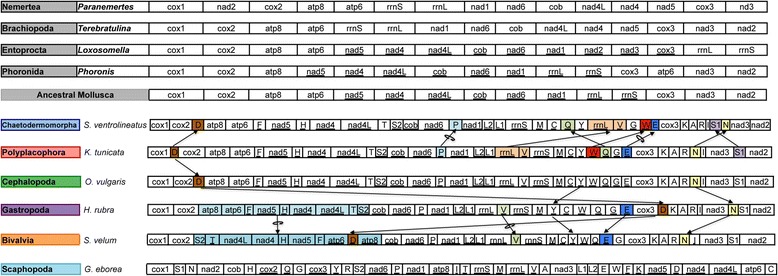
**Mitochondrial gene rearrangements between different mollusk classes.** Genes encoded by the opposite strand are underlined. Inversions (indicated by the circular arrow) and transpositions of protein-coding, tRNA and rRNA genes are depicted among the different taxa. Genes located in apomorphic arrangements are colored. The consensus protein-coding gene order of mollusks, and those of selected spiralian mt genomes are depicted. Genes have standard abbreviations and are not scaled to real length. S1, S2, L1, and L2 designate genes for those tRNAs recognizing the codons AGN, UCN, CUN, and UUR, respectively.

Compared with other mollusks, the mt gene order of *S. ventrolineatus* is very similar to those of Polyplacophora, Cephalopoda and basal lineages of Gastropoda (Vetigastropoda and Neritimorpha; but not of Patellogastropoda, [35]) (Figure 2). There are no differences in the relative position of protein-coding genes, but the *rrnL* gene is translocated in *Scutopus* mt genome with respect to the above-mentioned lineages (Figure 2). Other differences are found in the relative position of several tRNA genes; in particular *trnD*, *trnE*, and *trnN* are highly rearranged among mollusk lineages (Figure 2). The representative of an early-diverging lineage of Bivalvia (*Solemya velum*; Solemyoidea) also retains this gene order although a large inversion has occurred affecting a stretch including *atp8*-*atp6*-*nad5*-*nad4*-*nad4L* (Figure 2). Hence, our results allow us to propose a consensus ancestral mollusk gene order for mt protein-coding and rRNA genes (Figure 2). Only few translocation and inversion events are required to transform this consensus mollusk gene order into the genome organizations reported for other Spiralia, particularly Phoronida, Brachiopoda, Nemertea, and Entoprocta (Figure 2). The conversion to the genome organization of Annelida requires postulating additional translocations and inversions [37]. In Scaphopoda, the mt gene order is rather different to that of other mollusks, both in *Graptacme* (Figure 2) and *Siphonodentalium* [35]. In *Graptacme*, only the *nad6-trnP-nad1* and the *nad5-nad4-nad4L* arrangements are conserved, whereas gene pairs that are normally conserved in other metazoan mt genomes (e.g. *cox1*-*cox2* and *atp8*-*atp6*) are not found (Figure 2). New complete mt genome sequences of more (early-branching) lineages within Scaphopoda are needed in order to determine whether Scaphopoda ancestrally conformed or not to the consensus ancestral mt gene order of mollusks. Within Gastropoda and Bivalvia, highly derived mt genome organizations can be found due to extensive gene rearrangements [28,29].

### Phylogenetic relationships within Mollusca

Despite its popularity, phylogenetic inference based on complete mt genomes has important analytical challenges. In fact, reconstructing the monophyly and phylogenetic relationships of mollusks using solely mt genome data proved to be difficult. A first issue was finding the appropriate outgroup. Most recent phylogenies of spiralians place mollusks within the clade Trochozoa, which also includes annelids, nemerteans, brachiopods, and phoronids [38]. Among available mt genomes of spiralians, we selected representatives from one non-trochozoan spiralian (Entoprocta) and all trochozoan phyla. Another important problem was LBA due to among-lineage rate heterogeneity. Several ingroup lineages, notably Bivalvia, Scaphopoda, and Patellogastropoda (within Gastropoda), exhibited long branches that produced considerable inference biases (not shown), and they had to be excluded in further phylogenetic analyses. At least in several bivalves (families Donacidae, Hyriidae, Margaritiferidae, Mytilidae, Solenidae, Unionidae, and Veneridae), the occurrence of doubly uniparental inheritance (DUI) of the mt genome [39] has been proposed to cause higher substitution rates and thus faster saturation of the phylogenetic signal, hampering their use in reconstructing deep phylogenies. In addition, Neomeniomorpha and Monoplacophora could not be incorporated into the phylogenetic analyses because complete mt genomes are not available for these lineages thus far. Finally, due to the relatively high substitution rates of mtDNA, saturation is another major issue affecting the reconstruction of deep nodes based on mt markers. Here, we opted to analyze mt data at the amino acid level to reduce the effect of saturation.

**Figure 3:**
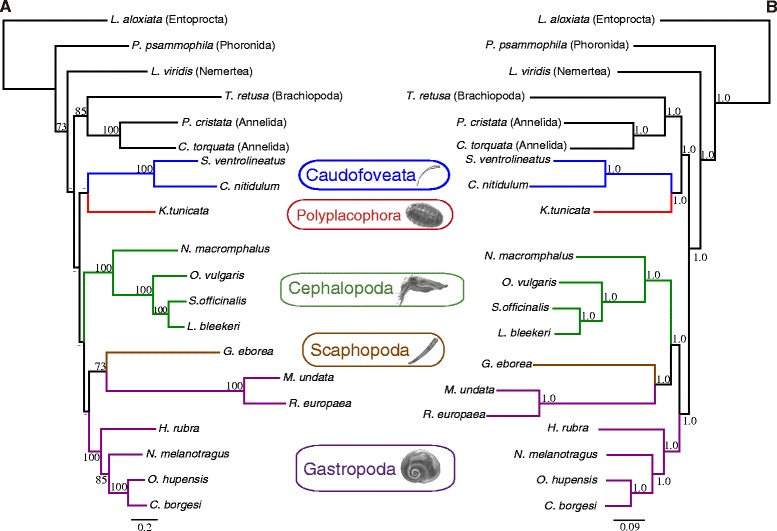
**Phylogenetic relationships of main mollusk lineages based on complete mt genome data.** The reconstructed ML **(A)** and BI **(B)** trees are depicted, inferred from a concatenated data set of mt protein coding genes at the amino acid level (see main text). Numbers above nodes correspond to bootstrap proportions **(A)** and Bayesian posterior probabilities **(B)**. Hyphens (-) indicate values below 70% bootstrap support.

The mt data set included 4870 and 2728 positions before and after sites of ambiguous positional homology were discarded. The fact that 44% of the initial length of the alignments was excluded by GBlocks suggests that mt genome amino acid sequence data have complex evolutionary patterns at higher levels of divergence, which would produce phylogenetic reconstruction biases if conflicting positions were not removed. ML (-lnL = 57186.85) and BI (arithmetic mean of the two runs, -lnL = 67264.29) arrived at relatively different trees (Figure 3). In ML, the monophyly of Mollusca is recovered with low bootstrap support whereas in BI, Brachiopoda + Annelida are recovered with maximal posterior probability support as sister group of Chaetodermomorpha + Polyplacophora, rendering Mollusca non-monophyletic. Within mollusks, the clades Aculifera and Conchifera received maximal posterior probability support in BI and low (<50%) bootstrap support in the ML analysis. The phylogenetic analyses failed to recover the monophyly of gastropods due to a LBA artifact (highly supported) between *Roboastra* + *Micromelo* (Heterobranchia) and Scaphopoda, both in ML and BI. This clade is the sister group of remaining gastropods with low bootstrap support in ML or of cephalopods with maximal posterior probability support in BI (Figure 3).

**Figure 4:**
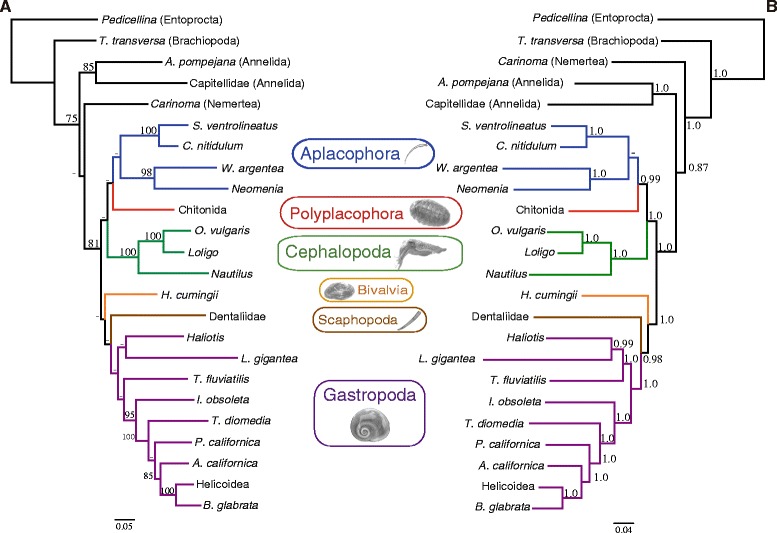
**Phylogenetic relationships of main mollusk lineages based on nuclear ribosomal protein data.** The reconstructed ML **(A)** and BI **(B)** trees are depicted, inferred from a concatenated data set of nuclear ribosomal proteins. Numbers above correspond to bootstrap proportions **(A)** and Bayesian posterior probabilities **(B)**. Hyphens (-) indicate values below 70% bootstrap support or 0.90 Bayesian posterior probabilities. Since our phylogenetic analyses were focused on the order level or above, and in order to maximize the completeness of the nuclear data set, sequences from related species/genera/families/superfamilies (for which there is strong evidence for their monophyly) were merged in some instances.

Recently, two phylogenies of Mollusca have been reconstructed based on large concatenated data sets of hundreds or thousands of nuclear gene fragments [24,25]. Here, we selected nuclear genes from those matrices that minimized missing data and maximized taxon coverage to resolve the question at hand. The partial deduced amino acid sequences of 13 nuclear ribosomal protein genes (see also [22]) were aligned for 5 outgroup taxa and main mollusk lineages (19 species). The concatenation of ribosomal proteins yielded an alignment of 2362 positions after removal of ambiguous sites. Phylogenetic analyses under ML (-lnL = 33338.02) and BI (arithmetic mean of the two runs, -lnL = 36866.21) recovered similar topologies that only differed on the relative position of the neritimorph *Theodoxus* (sister group of Caenogastropoda + Heterobranchia in ML and sister group of Vetigastropoda + Patellogastropoda in BI) and on the closest sister group to mollusks (a nemertean in ML and the annelids in BI) (Figure 4). In both ML and BI, Aplacophora and Aculifera were recovered as monophyletic (as in [24,25]), although with low support (except Aculifera in BI that has a posterior probability of 0.99). In contrast, both phylogenetic analyses failed to recover the monophyly of Conchifera because Cephalopoda was placed as sister group of Aculifera (with low support in ML but with maximal Bayesian posterior probability) (Figure 4). In both ML and BI, Bilvavia was recovered as sister group of Scaphopoda + Gastropoda in agreement with [25]. Interestingly, Scaphopoda and *Lottia* showed relatively long branches, which clearly classify them as rogue taxa, showing extremely fast evolutionary rates for both mt and nuclear genes; [33,40]. The reconstructed trees based on the nuclear data set improved previous results obtained from ribosomal nuclear protein [22] and the *18S* rRNA gene [40], which recovered Cephalopoda as the sister group of Chaetodermomorpha or Neomeniomorpha, respectively. However, our reconstructed trees resemble those based on housekeeping genes [23,40] that also placed Cephalopoda a sister group to Aculifera. The odd placement of Cephalopoda here and in the above-mentioned studies [22,23,40] contradicts the general agreement placing cephalopods within Conchifera [9]. This might be a tree reconstruction artifact produced by LBA biases generated by the inclusion of relatively distantly related spiralian phyla in the outgroup.

**Figure 5:**
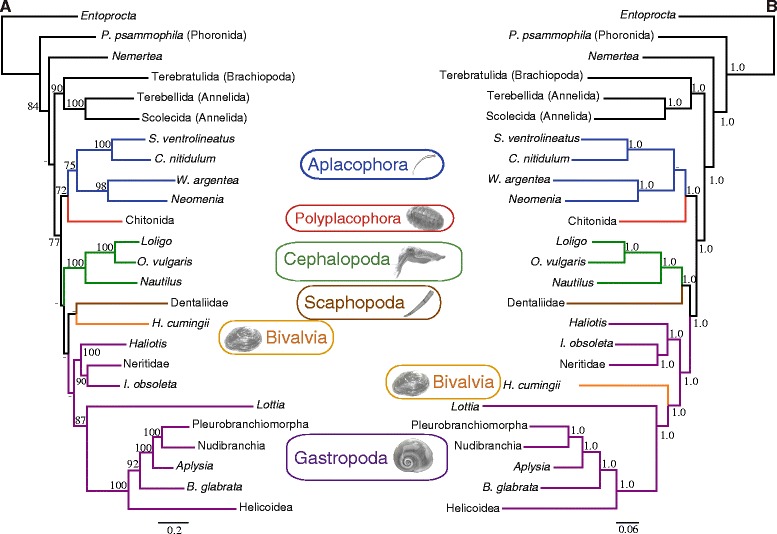
**Mitochondrial and nuclear combined evidence on mollusk phylogeny.** The reconstructed ML **(A)** and BI **(B)** trees are depicted. Numbers above nodes correspond to bootstrap proportions **(A)** and Bayesian posterior probabilities **(B)**. Hyphens (-) indicate values below 70% bootstrap support or 0.90 Bayesian posterior probabilities. Since our phylogenetic analyses were focused on the order level or above, and in order to maximize the completeness of the combined data set, sequences from related species/genera/families/superfamilies (for which there is strong evidence for their monophyly) were merged in some instances.

Although with a different lineage sampling and species representation, the mt and nuclear data sets rendered rather congruent trees with several nodes in common. Therefore, we concatenated the nuclear and mt data sets. The phylogenetic analyses of the combined (mt and nuclear) data set (5032 sites after removal of ambiguous positions) under ML (-lnL = 101714.93) and BI (arithmetic mean of the two runs, -lnL = 116220.97) arrived at very similar topologies that only differed in the relative position of Bivalvia and Scaphopoda (Figure 5). The monophyly of Aplacophora, Aculifera and Conchifera were recovered both by ML and BI (Figure 5). The corresponding nodes received relatively high support except Conchifera in ML and Aplacophora in BI (Figure 5). In ML and BI, Cephalopoda and Cephalopoda + Scaphopoda were recovered as sister group of the remaining analyzed conchiferan classes, respectively (Figure 5). In ML, Gastropoda was recovered as sister group of Bivalvia + Scaphopoda, whereas in BI Bivalvia was placed nested within Gastropoda, rendering the latter non-monophyletic (Figure 5). These results agree with most recent phylogenomic studies [24,25] in the basal position of Cephalopoda within Conchifera, and favor either a close relationship of Gastropoda and Bivalvia to the exclusion of Scaphopoda [24] or the classical Diasoma hypothesis uniting Bivalvia and Scaphopoda [9]. It is noteworthy that the reconstructed phylogeny based on the combined data set differs from the nuclear-based tree indicating that addition of mt data has a significant (and distinct) contribution to the overall phylogenetic inference. Furthermore, previously encountered LBA artifacts when mt data was analyzed alone (related to Scaphopoda, Bivalvia and *Lottia*) were ameliorated in the combined analysis.

## Conclusions

Reconstructing the monophyly and internal phylogenetic relationships of Mollusca based on molecular data has been challenging over the years. Earlier studies were mostly based on partial gene sequences with insufficient informative characters to reconstruct robust and resolved trees [18]. Recent phylogenomic studies [24,25] based on concatenated nuclear genes have produced well-resolved trees. However, comparison of these trees showed some contradicting but highly supported nodes. Another alternative is to investigate phylogenetic information contained in complete mt genomes. A first attempt to use whole mt genome data for clarifying the relative phylogenetic position of mollusks within bilaterians was not satisfying [35]. Our phylogenetic analyses reveal that the use of mt genome data for reconstructing the internal phylogenetic relationships of mollusks is flawed by the existence of high heterogeneity of evolutionary rates among lineages. In particular, the mt genomes of Bivalvia and *Lottia* introduce detrimental LBA biases. After removing taxa that exhibit long branches in the phylogenetic analyses, mt data are capable of recovering the monophyly of each Aculifera, and Conchifera, in agreement with nuclear data (which additionally recover the monophyly of Aplacophora). Moreover, our analyses indicate that the phylogenetic performance of mt and nuclear data improves when both are combined. At present, the complete mt genomes of several important mollusk lineages (namely Neomeniomorpha and Monoplacophora) are still missing, and the possibility of finding mt genomes with lower substitution rates in Bilvalvia and Patellogastropoda needs to be further explored. It is foreseeable that the addition of these additional mt genomes will improve phylogenetic analyses. In parallel, gapped regions in nuclear genomic data sets will be increasingly reduced allowing in combination with mt data the reconstruction of a robust tree of Mollusca. Such a robust phylogenetic hypothesis has been long-needed as the framework for evolutionary comparative studies within this highly diversified metazoan phylum.

## Methods

### DNA extraction, PCR amplification, cloning and sequencing

Several specimens of *Scutopus ventrolineatus* Salvini-Plawen, 1968 (Mollusca; Chaetodermomorpha) were collected in March 2010 close to Bergen, in the Norwegian west coast. Total genomic DNA of a single specimen was isolated following standard phenol-chloroform extraction procedures [41]. Two fragments corresponding to partial mt *cox1cox2* genes were PCR -amplified using appropriate universal primer pairs: LCOI 1490, HCO 2198 [42] and cox2F1, cox2R1 [43], respectively. PCR reactions contained 2.5 μl of 10x Ex *Taq* Buffer, 2 μl of dNTP Mixture (2.5 mM each), 1.5 μl of each primer, 0.5 μl of template DNA, 0.16 μl Ex *Taq* Hot Start DNA polymerase (5 units/μl; TaKaRa Bio Inc., Otsu, Shiga, Japan), and sterilized distilled water up to 25 μl. The following temperature profile was used: an initial denaturing step at 98°C for 2 min; 35 cycles of denaturing at 98°C for 10 s, annealing at 50°C for 30 s, and extending at 72°C for 1 min; and a final extending step at 72°C for 1 min. PCR products were purified by ethanol precipitation, and sequenced in an automated DNA sequencer ABI PRISM 3700 using the BigDye Terminator v3.1 cycle-sequencing kit (Applied Biosystems; Foster City, CA, USA), and PCR primers.

Newly determined partial sequences of mt *cox1* and *cox2* were used to design two pairs of specific primers for long range PCR amplification (SVCOX1.F: 5'-TTT TTG ACC CTG CTG GAG GTG GAG AC-3'; SVCOX1.R: 5'-AGA GGG GGG TAT ACA GTC CAC CCA GTC-3'; SVCOX2.F: 5'-TCC CAG CAT TGG GAG TAA AAG CCG AC-3'; SVCOX2.R: 5'-CTC CGC AGA TTT CTG AAC ATT GAC CA-3'). The full mt genome was amplified in two overlapping fragments of 13003 bp and 1771 bp, respectively, using the TaKaRa LA-PCR kit (TaKaRa Bio Inc., Otsu, Shiga, Japan). PCR amplifications were carried out in 50 μl reactions containing 5 μl of 10x LA Buffer II (Mg^+2^ plus), 8 μl of dNTP Mixture (2.5 mM each), 1 μl of each primer, 0.1 μl of template DNA and 0.5 μl Taq DNA polymerase (5 units/μl). The following temperature profile was used: an initial denaturing step at 94°C for 1 min; 45 cycles of denaturing at 98°C for 10 s, annealing at 57°C for 30 s, and extending at 68°C for 1 min per Kb; and a final extending step at 68°C for 10 min. Sequencing of the two long PCR fragments was achieved with the shotgun technique using the TOPO-Shotgun subcloning Kit (Invitrogen; Life Technologies, Paisley, UK). Random clone libraries were constructed from purified PCR products by shearing them into fragments of 1–3 Kb in size, by repairing fragment ends to form blunt-ends, and by cloning blunt-ended fragments into pCR 4Blunt-TOPO vectors. Clones were sequenced in an automated DNA sequencer ABI PRISM 3700 using the BigDye Terminator v3.1 cycle-sequencing kit (Applied Biosystems; Foster City, CA, USA), and M13 forward and reverse universal primers.

### Genome assembly and annotation

The complete mt genome was assembled into a single contig from the shotgun clone sequences using Sequencher v. 5.0.1 (Gene Codes Co.; Ann Arbor, MI, USA). The mt genome was annotated using the MITOS [44] and DOGMA [45] webservers. Briefly, protein-coding genes were annotated by identification of their open reading frames and similarity searches against other reported mollusk mt genomes. Ribosomal RNA genes were identified by sequence comparison with other reported mollusk mt genomes, and assumed to extend to the boundaries of adjacent genes. Transfer RNA genes were identified using tRNAscan-SE v. 1.21 [46] and ARWEN v. 1.2 [47], which can infer cloverleaf secondary structures of the corresponding gene products. The complete mt genome sequence reported in this paper has been deposited at NCBI GenBank under accession number KC757645.

The gene order of the mt genome of *S. ventrolineatus* was compared to the following mollusk mt genomes: *Chaetoderma nitidulum* (Chaetodermomorpha; Dreyer and Steiner, unpublished), *Katharina tunicata* (Polyplacophora; [31]), *Graptacme eborea* (Scaphopoda; [34]), *Octopus vulgaris* (Cephalopoda; [27]), *Solemya velum* (Bivalvia; [48]), and *Haliotis rubra* (Gastropoda; [49]). The following spiralian mt genomes were also included in the gene order comparisons: *Paranemertes* cf. *peregrina* (Nemertea; [50]), *Terebratulina retusa* (Brachiopoda; [51]), *Loxosomella aloxiata* (Entoprocta; [52]), and *Phoronis architecta* (Phoronida; [53]).

### Data sets and sequence alignment

Amino acid sequences derived from the 13 mt protein-coding genes were used to assemble the mt data set, which included 14 representatives of the main extant mollusk lineages and 6 species representing several metazoan phyla other than Mollusca (see Additional file 1: Table S1). Similarly, a nuclear data set was constructed with the deduced amino acid sequences of the genes coding for 40S ribosomal proteins S8 and S15, and 60S ribosomal proteins L3, L4, L5, L6, L7, L8, L10a, L16_L10, L17, L18a, and L32. These genes were extracted from Kocot et al. (2011) and were selected among available genes because they minimized missing data. This nuclear data set included five species representing several metazoan phyla other than Mollusca and 19 representatives of the main extant mollusk lineages (see Additional file 1: Table S2). A third data set was constructed combining the mt and nuclear data sets. The three data sets were designed to test specifically the monophylies of Mollusca and Aculifera. Since our phylogenetic analyses were focused on the order level or above, and in order to maximize the completeness of the nuclear and combined data sets, sequences from related species/genera/families/superfamilies (for which there is strong evidence for their monophyly) were merged in some instances (see Additional file 1: Table S2; [24]). Note that the original study from which nuclear data was extracted [24] already merged closely related species to maximize gene coverage. The species showing the shortest branches were selected as representatives of the different lineages in the three data sets.

Deduced amino acid sequences of the different mt and nuclear protein-coding genes were downloaded from GenBank and aligned separately using MAFFT v. 7 [54] with default settings. Ambiguously aligned positions were removed using Gblocks, v. 0.19b [55] with default settings.

### Phylogenetic analyses

Alignment format conversions were performed using the ALTER webserver [56]. For the three analyzed data sets, best-fit partition schemes and models of amino acid replacement were identified using the Akaike information criterion (AIC; [57]) as implemented in PartitionFinderProtein [58]. For the mt data set, we tested the following *a priori* partition schemes: (1) all genes combined; (2) genes by functional group (*atp*, *cox*, *nad*, *cob)*; (3) all genes separately except *atp8/atp6* and *nad4L/nad4,* and (4) all genes independently. For the nuclear data set we tested (1) all genes combined, (2) by functional group (*40S* and *60S* genes), and (3) all independent. For the combined data set, we tested all above-mentioned partition schemes. The AIC favored independent gene partitions in the nuclear and the combined data sets, whereas the best partition scheme for the mt data sets was that with all genes analyzed separately except for *atp8/atp6* and *nad4L/nad4*. The resulting best-fit models for each partition are shown in Additional file 1: Table S3.

Phylogenetic relationships were inferred using maximum likelihood (ML) and Bayesian inference (BI). ML analyses were conducted with RAxML v. 7.0.4 [59] using the rapid hill-climbing algorithm. For BI, we used MrBayes v. 3.1.2 [60] running two independent analyses, each consisting in four simultaneous MCMC (Markov chain Monte Carlo) for 10 million generations, sampling every 1,000 generations, and discarding the first 25% generations as burnin (as judged by plots of ML scores and low SD of split frequencies) to prevent sampling before reaching stationarity of Markov chains. Support for internal branches was evaluated by non-parametric bootstrapping [61] with 1,000 replicates (ML) and by posterior probabilities (BI).

## Additional file

Additional file 1: Figure S1. Comparison of mitochondrial gene orders of *Scutopus ventrolineatus* and *Chaetoderma nitidulum*. **Table S1.** Complete mitochondrial genomes used in the phylogenetic analyses. **Table S2.** Fragments of nuclear ribosomal proteins used in phylogenetic analyses. **Table S3.** Best-fit partitions and models selected by Protein Partition Finder.

## Abbreviations

*atp6-8*: ATP synthase subunits 6-8
BI: Bayesian inference
bp: Base pairs
*cob*: Cytochrome *b*
*cox1-3*: Cytochrome c oxidase subunits 1-3
LBA: Long branch attraction
ML: Maximum likelihood
mt: Mitochondrial
*nad1-6*: *4 L*, NADH dehydrogenase subunits 1-6, 4 L
PCR: Polymerase chain reaction
rRNA: Ribosomal RNA
*rrnL*: Large ribosomal RNA
*rrnS*: Small ribosomal RNA
tRNA: Transfer RNA
*trnX*: Transfer RNA for amino acid X (denoted by the one-letter IUPAC symbol)
