# Supplementary material for: The complete mitochondrial genome of Scutopus ventrolineatus (Mollusca: Chaetodermomorpha) supports the Aculifera hypothesis
Source: BMC Evol Biol. 2014 Sep 25;14:197. doi: 10.1186/s12862-014-0197-9 (PMC4189740; doi:10.1186/s12862-014-0197-9)
Supplement: Additional file 1: Figure S1. — Comparison of mitochondrial gene orders of Scutopus ventrolineatus and Chaetoderma nitidulum. Table S1. Complete mitochondrial genomes used in the phylogenetic analyses. Table S2. Fragments of nuclear ribosomal proteins used in phylogenetic analyses. Table S3. Best-fit partitions and models selected by Protein Partition Finder. [file 12862_2014_197_MOESM1_ESM.pdf]

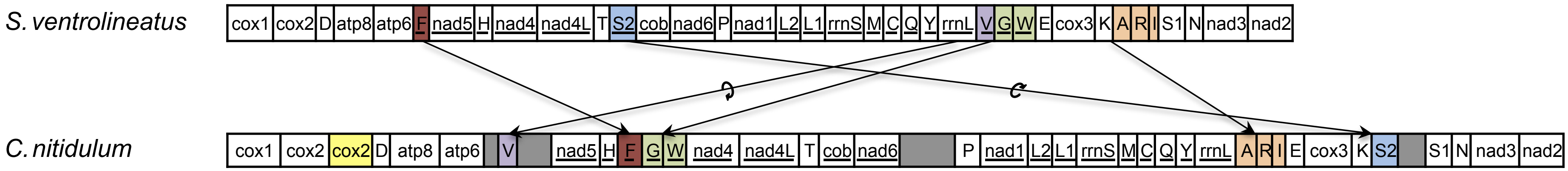

Appendix. Figure 1
Mitochondrial gene orders of *Scutopus ventrolineatus* and *Chaetoderma nitidulum*. Inversions (indicated by the circular arrows) and transpositions of protein coding, tRNA and rRNA genes are depicted among the two taxa. Genes encoded by the opposite strand are underlined. Genes located in apomorphic arrangements are colored. The biggest non coding regions (>300pb) are colored in grey. S1, S2, L1, and L2 designate genes for those tRNAs recognizing the codons AGN, UCN, CUN, and UUR, respectively.

Table 1. Complete mitochondrial genomes used in the phylogenetic analyses.

Taxon		Species	NCBI Accession No.	Reference
**GASTROPODA**				
HETEROBRANCHIA				
	Hygrophila: Planorboidea	*Biomphalaria glabrata*[1]	NC_005439	DeJong et al., 2004
	Nudipleura: Nudibranchia: Doridina	*Roboastra europaea*	NC_004321	Grande et al., 2002
	Nudipleura: Pleurobranchomorpha	*Berthellina* sp.[1]	NC_015091	Medina et al. 2011
	Architectibranchia	*Micromelo undata*	NC_015106	Medina et al., 2011
	Euopisthobranchia: Aplysiomorpha	*Aplysia dactylomela*[1]	NC_015088	Medina et al., 2011
CAENOGASTROPODA				
	Neogastropoda: Conoidea	*Conus borgesi*	NC_013243	Cunha et al., 2009
	Neogastropoda: Buccionidea	*Ylianassa obsoleta*[1]	NC_007781	Simison et al. 2006
	Littorinimorpha: Rissooidea	*Oncomelania hupensis*	NC_013073	Li and Zhou, 2009*
VETIGASTROPODA				
	Haliotoidea	*Haliotis rubra*	NC_005940	Maynard et al., 2005
PATELLOGASTROPODA				
	Lottioidea	*Lottia digitalis*[1]	NC_007782	Simison et al., 2006
NERITIMORPHA				
	Neritidae	*Nerita melanotragus*	GU810158	Castro and Colgan, 2010
**BIVALVIA**				
	Unionoidea	*Hyriopsis cumingii*[1]	NC_011763	Zheng and Li, 2008*
**CEPHALOPODA**				
	Decapodiformes: Sepiida	*Sepia officinalis*	NC_007895	Akasaki et al., 2006
	Decapodiformes: Teuthida	*Loligo bleekeri*	NC_002507	Tomita et al., 2002
	Octopodiformes: Octopoda	*Octopus vulgaris*	NC_006353	Yokobori et al., 2004
	Nautiloidea	*Nautilus macromphalus*	NC_007980	Boore, 2006

**POLYPLACOPHORA**				
	Chitonida	*Katharina tunicata*	NC_001636	Boore and Brown, 1994
**SCAPHOPODA**				
	Dentaliidae	*Graptacme eborea*	NC_006162	Boore et al., 2004
**CHAETODERMOMORPHA**				
	Chaetodermatida	*Chaetoderma nitidulum*	NC_013846	Dreyer and Steiner, 2010*
	Limifossorimorpha	*Scutopus ventrolineatus*	KC_757645	This paper
**ANNELIDA**				
	Scolecida; Maldanidae	*Clymenella torquata*	NC_006321	Jennings and Halanych, 2005
	Terebellida; Terebellidae	*Pista cristata*	NC_011011	Zhong et al., 2008
**NEMERTEA**				
	Heteronemertea	*Lineus viridis*	NC_012889	Podsiadlowski et al, 2009
**BRACHIOPODA**				
	Terebratulida	*Terebratulina retusa*	NC_000941	Stechmann and Schlegel, 1999
**PHORONIDA**				
	Phoronidae	*Phoronis psammophila*	AY368231	Helfenbein and Boore, 2004
**ENTOPROCTA**				
	Loxosomatidae	*Loxosomella aloxiata*	NC_010432	Yokobori et al., 2008

* Unpublished
[1]used only the combined data set

References

Akasaki, T., Nikaido, M., Tsuchiya, K., Segawa, S., Hasegawa, M., Okada, N., 2006. Extensive mitochondrial gene arrangements in coleoid Cephalopoda and their phylogenetic implications. Mol. Phylogenet. Evol. 38, 648-658.

Boore, J., 2006. The complete sequence of the mitochondrial genome of *Nautilus macromphalus* Mollusca: Cephalopoda. BMC Genomics 7, 182.

Boore, J.L., Brown, W.M., 1994. Complete DNA Sequence of the Mitochondrial Genome of the Black Chiton, *Katharina tunicata*. Genetics 138, 20.

Boore, J.L., Medina, M., Rosenberg, L.A., 2004. Complete Sequences of the Highly Rearranged Molluscan Mitochondrial Genomes of the Scaphopod *Graptacme eborea* and the Bivalve *Mytilus edulis*. Mol. Biol. Evol 21.

Castro, L.R., Colgan, D.J., 2010. The phylogenetic position of Neritimorpha based on the mitochondrial genome of *Nerita melanotragus* Mollusca: Gastropoda. Mol. Phylogenet. Evol. 57, 918-923.

Cunha, R., Grande, C., Zardoya, R., 2009. Neogastropod phylogenetic relationships based on entire mitochondrial genomes. BMC Evol. Biol. 9, 210.

DeJong, R., Emery, A., Adema, C., 2004. The mitochondrial genome of Biomphalaria glabrata Gastropoda: Basommatophora, intermediate host of *Schistosoma mansoni*. J. Parasitol. 90, 991-997.

Dreyer, H., Steiner, G., 2004. The complete sequence and gene organization of the mitochondrial genome of the gadilid scaphopod *Siphonondentalium lobatum* Mollusca. Mol. Phylogenet. Evol. 31, 605-617.

Grande, C., Templado, J., Cervera, J.L., Zardoya, R., 2002. The complete mitochondrial genome of the nudibranch Roboastra europaea Mollusca: Gastropoda supports the monophyly of opisthobranchs. Mol. Biol. Evol. 19, 1672-1685.

Helfenbein, K. G. and Boore, J. L., 2004. The mitochondrial genome of *Phoronis architecta*. Comparisons demonstrate that phoronids are lophotrochozoan protostomes. Mol. Biol. Evol. 21, 153-157.

Jennings, R.M., Halanych, K.M. 2005. Mitochondrial Genomes of *Clymenella torquata* (Maldanidae) and *Riftia pachyptila* (Siboglinidae): Evidence for conserved gene order in annelida. Mol. Biol. Evol. 22, 210-222

Maynard, B., Kerr, L., McKiernan, J., Jansen, E., Hanna, P., 2005. Mitochondrial DNA sequence and gene organization in Australian backup abalone *Haliotis rubra* leach. Mar Biotechnol NY 7, 645 - 658.

Medina, M., Lal, S., VallËs, Y., Takaoka, T.L., Dayrat, B.A., Boore, J.L., Gosliner, T., 2011. Crawling through time: Transition of snails to slugs dating back to the Paleozoic, based on mitochondrial phylogenomics. Mar. Genom. 4, 51-59.

Podsiadlowski,L., Braband,A., Struck,T.H., von Dohren,J. And Bartolomaeus,T., 2009. Phylogeny and mitochondrial gene order variation in Lophotrochozoa in the light of new mitogenomic data from Nemertea. BMC Genomics 10, 364.

Simison, W., Lindberg, D., Boore, J., 2006. Rolling circle amplification of metazoan mitochondrial genomes. Mol. Phylogenet. Evol. 39, 562-567.

Stechmann, A. and Schlegel, M., 1999. Analysis of the complete mitochondrial DNA sequence of the brachiopod *Terebratulina retusa* places Brachiopoda within the protostomes. Proc. R. Soc. Lond., B, Biol. Sci. 266, 2043-2052.

Tomita, K., Yokobori, S.-I., Oshima, T., Ueda, T., Watanabe, K., 2002. The Cephalopod *Loligo bleekeri* Mitochondrial Genome: Multiplied Noncoding Regions and Transposition of tRNA Genes. J. Mol. Evol. 54, 486-500.

Yokobori, S.-i., Fukuda, N., Nakamura, M., Aoyama, T., Oshima, T., 2004. Long-Term Conservation of Six Duplicated Structural Genes in Cephalopod Mitochondrial Genomes. Mol. Biol. Evol 21, 2034-2046.

Yokobori,S., Iseto,T., Asakawa,S., Sasaki,T., Shimizu,N., Yamagishi,A., Oshima,T. and Hirose,E. 2008. Complete nucleotide sequences of mitochondrial genomes of two solitary entoprocts, *Loxocorone allax* and *Loxosomella aloxiata*: implications for lophotrochozoan phylogeny. Mol. Phylogenet. Evol. 47, 612-628.

Zhong,M., Struck,T.H. and Halanych,K.M. 2008. Phylogenetic information from three mitochondrial genomes of Terebelliformia (Annelida) worms and duplication of the methionine tRNA. Gene 416, 11-21.

Table 2. Fragments of nuclear ribosomal proteins used in phylogenetic analyses.

Taxon		*Nuclear ribosomal proteins*[1]											
		40S		*60S*									
		S8	*S15*	*L3*	*L4*	*L5*	*L6*	*L8*	*L10a*	*L16_L10*	*L17*	*L18a*	*L32*
Pleurobranchaea californica	*Gastropoda: Heterobranchia*	+	+	+	+	+	+	+	+	+	+	+	+
Aplysia californica	*Gastropoda: Heterobranchia*	+	+	+	+	+	+	+	+	+	+	-	+
Biomphalaria glabrata	*Gastropoda: Heterobranchia*	+	+	+	+	+	+	+	+	+	-	+	-
Tritonia diomedia	*Gastropoda: Heterobranchia*	+	+	-	-	+	+	+	+	+	+	+	+
Helicoidea	*Gastropoda: Heterobranchia*	+	-	+	+	-	-	+	+	+	-	+	+
Ilyanassa obsoleta	*Gastropoda: Caenogastropoda*	+	-	-	+	+	+	-	+	+	-	+	+
Theodoxus fluviatilis	*Gastropoda: Neritimorpha*	+	+	+	+	+	-	+	+	+	+	+	+
Haliotis	*Gastropoda: Vetigastropoda*	+	+	+	+	+	+	+	+	+	+	+	-
Lottia gigantea	*Gastropoda: Patellogastropoda*	+	+	+	+	+	+	+	+	+	+	+	-
Dentaliidae	*Scaphopoda: Dentaliida*	+	+	+	+	+	-	+	+	+	+	-	+
Hyriopsis cumingii	*Bivalvia: Unionoidea*	+	+	+	+	+	+	-	-	+	+	+	+
Loligo	*Cephalopoda: Decapodiformes*	+	+	+	+	+	+	+	+	+	+	+	+
Octopus vulgaris	*Cephalopoda: Octopodiformes*	+	-	+	+	+	+	+	+	+	+	+	+
Nautilus	*Cephalopoda: Nautiloidea*	-	+	-	+	+	+	+	+	+	+	+	+
Chitonida	*Polyplacophora*	+	-	+	+	-	+	+	+	+	+	+	+
Scutopus ventrolineatus	*Chaetodermomorpha*	+	+	+	+	+	+	-	+	+	-	-	+
Chaetoderma nitidulum	*Chaetodermomorpha*	+	+	-	-	+	+	+	+	+	+	+	+
Neomenia	*Neomeniomorpha*	-	+	-	-	-	+	-	+	+	+	+	-
Wirenia argentea	*Neomeniomorpha*	+	+	+	+	+	+	+	+	+	-	+	+
Capitellidae	*Annelida*	+	+	+	+	+	+	+	+	+	+	+	-
Alvinella pompejana	*Annelida*	+	+	+	+	+	+	+	-	+	+	+	+
Carinoma	*Nemertea*	+	+	+	-	-	+	-	+	+	+	+	+
Terebratalia transversa	*Brachiopoda*	+	+	+	+	+	+	+	-	-	+	+	+
Pedicellina	*Entoprocta*	-	-	-	+	-	-	-	-	+	-	+	-
No. Amino acids		*204*	*134*	*244*	*221*	*246*	*151*	*244*	*197*	*203*	*121*	*280*	*117*

[1] *Kocot, K.M., Cannon, J.T., Todt, C., Citarella, M.R., Kohn, A.B., Meyer, A., Santos, S.R., Schander, C., Moroz, L.L., Lieb, B., Halanych, K.M., (2011) Phylogenomics reveals deep molluscan relationships. Nature 477: 452-456.*

Table 3. Output of the Protein Partition Finder

Data set	Best Model	alpha	pinvar
**MITO amino acids**	**best partition: per group (*atp, cox, cob, nad*); -lnL= 67264.29**		
atp	mART	0.87281	
cox	LG	0.434848	
cob	mART	0.642182	
nad	mART	0.655589	
**NUC amino acids**	**best partition: all genes separated; -lnL= 36866.21**		
40S_S8	LG	0.81703	
40S_S15	JTT	0.675925	
60S_L3	LG	0.593494	
60S_L4	LG	0.705006	
60S_L5	LG	1.253	0.268898
60S_L6	LG	0.514199	
60S_L8	LG	0.530343	
60S_L10a	LG	1.154	0.265532
60S_L16_L10	LG	0.615296	0.241674
60S_L17	JTT	0.843745	0.157126
60S_L18a	Blosum62	1.049	
60S_L32	LG	0.864881	0.225493
**Combined (MITO+NUC)**	**best partition: *atp*6/8 and *nad*4/4L, remaining separated; -lnL= 178340.19**		
atp6/8	mART	0.747941	
cox1	LG	0.473613	
cox2	mART	0.675584	
cox3	mART	0.577307	
cob	mART	0.588785	
nad1	mART	0.586762	

nad2	mART	0.63904
nad3	mART	0.960463
nad4/4L	LG	0.631425
nad5	LG	0.487988
nad6	mART	0.764513
40S_S8	LG	0.752367
40S_S15	JTT	0.521373
60S_L3	LG	0.555502
60S_L4	LG	0.761228
60S_L5	LG	0.610006
60S_L6	LG	0.60341
60S_L8	LG	0.462332
60S_L10a	LG	0.504528
60S_L16_L10	LG	0.372527
60S_L17	LG	0.440494
60S_L18a	Blosum62	0.959612
60S_L32	LG	0.41837
